# 3D Carbon Nanonetwork Coated Composite Electrode with Multi-Heteroatom Doping for High-Rate Vanadium Redox Flow Batteries

**DOI:** 10.3390/polym14235269

**Published:** 2022-12-02

**Authors:** Wei Ling, Xiongwei Wu, Funian Mo

**Affiliations:** 1 School of Materials Science and Engineering, Harbin Institute of Technology (Shenzhen), Shenzhen 518055, China; 2 School of Chemistry and Materials Science, Hunan Agricultural University, Changsha 410128, China

**Keywords:** graphite fibers electrodes, 3D nanonetwork, multi-heteroatom doping, high-rate property, vanadium redox flow batteries

## Abstract

With the advantages of benign mechanical property, electrochemical stability, and low cost, graphite fibers (GFs) have been widely used as electrodes for vanadium redox flow batteries (VRFBs). However, GFs usually possess inferior electrochemical activity and ion diffusion kinetics for electrode reaction, vastly limiting their application in VRFBs. Here, a 3D carbon nanonetwork coated GFs with multi-heteroatom doping was constructed for application in VRFBs via low temperature polymerization between linear polymer monomer and phytic acid, and subsequent carbonization (900 °C) on the GFs (GF@PCNs-900). Benefiting from the 3D structural features and multi-heteroatom doping (O, N and P), the composite electrode displayed sufficient diffusion of vanadium ions, rapid electron conduction, and highly enhanced electrochemical activity of reactive site on the electrodes. As a result, the GF@PCNs-900 delivered a high discharge capacity of 21 Ah L^−1^ and energy efficiency of above 70% with extraordinary stability during 200 cycles at 200 mA cm^−2^. Even at a huge current density of 400 mA cm^−2^, the GF@PCNs-900 still maintained a discharge capacity of 5.0 Ah L^−1^, indicating an excellent rate of performance for VRFBs. Such design strategy opens up a clear view for further development of energy storage field.

## 1. Introduction

With the continuous progress of the global economy, fossil energy has been consumed excessively and brings about severe issues such as energy crises and environmental pollution, hindering the sustainable development of human society [1,2]. Thus, the exploitation of renewable and clean energy sources will become the ultimate form of energy revolution in the future due to their inexhaustible and environmentally friendly properties. However, these renewable and clean energy sources are usually enslaved to the effect of region and climate, and their energy output shows features of intermittency and volatility [3,4]. As supporting facilities, energy storage systems play an indispensable role in the practical application of renewable and clean energy sources. Compared with other energy storage systems, vanadium redox flow batteries (VRFBs) possess the great advantages of desirable safety, low cost of maintenance, durable life, and environmentally friendliness [5,6,7]. In addition, due to applying the same vanadium element as positive and negative reaction species, the VRFBs display a negligible cross-contamination effect between cathode and anode electrolytes in comparison with other flow batteries [8,9,10], such as zinc-iodine flow batteries [11,12], zinc-bromine flow batteries [13,14], and iron–based flow batteries [15,16,17].

As a key component, the electrode materials offer the reaction zone of vanadium redox, and their electrocatalytic activity is deeply related to the energy efficiency (EE) and rate performance of VRFBs [18,19,20]. The redox reaction on the electrodes can be described as follows:

Catholyte: VO^2+^ + 2H^+^ ↔ VO_2_^+^ + H_2_O − e^−^ (1)

E_0_ = 1.0 V vs. SHE

Anolyte: V^3+^ + e^−^ ↔ V^2+^ (2)

E_0_ = −0.26 V vs. SHE

The processes were initially investigated by Sun and Skyllas-Kazacos [21,22]. Due to low cost, superior corrosion resistance, and electrochemical stability, graphite fibers (GFs) have been widely applied as electrode materials in VRFBs. However, the original GFs usually present poor electrochemical activity and sluggish kinetics of electron and ion transport, largely restricting the rate performance and cycling stability of VRFBs. To enhance the electrochemical performance of GFs, various methods, including surface functional groups [23,24,25,26,27,28], metal and metallic oxides deposition [29,30,31], element doping [32,33,34], and functional carbon nanomaterial [35,36,37] have been applied to modify the composite electrodes for VRFBs. Firstly, the surface functionalization, such as acid treatment, heat treatment electrochemical oxidation, and oxygen plasma treatment, mainly introduce oxygen-containing functional groups on the electrode surface, and the abundant oxygen-containing functional groups can effectively improve the hydrophily of GFs [38,39], which prompts the sufficient wettability between electrolyte and GFs, and enhances the redox reaction of vanadium ions. Due to better electrocatalytic activity or electronic conductivity, metal and metallic oxides deposition has been used to elevate the electrochemical performance of GFs [40,41,42], but their worse stability and expensive cost will challenge their practical application. Owing to the difference in electronegativity and atomic size with substrate atoms, hetero-atoms doping can break the Π bond conjugated system among carbon atoms in GFs, and then bring about defect sites in the graphite carbon skeleton, which contribute to boosting the electrochemical activity of composite electrodes [43,44,45]. Moreover, the element doped GFs still maintain stable electrochemical performance during long-term cycling because the hetero-atoms in the form of covalent bond are introduced into composite electrodes [46]. The functional carbon nanomaterials, such as biomedical carbon [7,33], graphene [47], reduced graphene oxide (rGO) [48], carbon nanotube (CNTs) [49] and carbon nanofiber (CNFs) [50], are also used to modify GFs electrodes for VRFBs. Attributing to high surface area and excellent electronic conductivity, the functional carbon nanomaterial can accelerate charge transfer and ion diffusion during vanadium ions redox reaction, which is conductive to stimulate a high rate performance and energy efficiency of VRFBs [51]. Although various strategies have made great progress in the electrode research, a single way is hard to satisfy overall performance requirements of VRFBs. Thus, the optimal integration of multiple strategies for GFs is of great significance to elevate the electrochemical property of VRFBs.

Herein, this work prepared a 3D carbon nanonetwork coated GFs combined with multi-heteroatom doping (O, N and P) by cross-linking polymerization between linear polymer monomer and phytic acid, and finally carbonization on the GFs (GF@PCNs). In the polymerization process, the phytic acid molecule served as a cross-linking agent, not only to participate in the construction of 3D nanonetwork structure, but also to achieve the effective doping of oxygen and phosphorus element. On the one hand, the 3D nanonetwork contributed to the sufficient diffusion of vanadium ions and rapid electron conduction along the GFs surface. On the other hand, the multi-heteroatom doping highly improves the electrocatalytic activity of composite electrodes toward vanadium ions redox reaction. Due to the synergistic effect, the VRFBs based on GF@PCNs exhibited higher energy efficiency of above 70% during 200 cycles at 200 mA cm^−2^ and excellent rate performance for discharge capacity of 5.0 Ah L^−1^ at huge current density of 400 mA cm^−2^.

## 2. Experimental

### 2.1. Preparation of GF@PCNs

Firstly, the phytic acid solution (Aladdin, Shanghai, China) of 70 wt% was diluted to 6 wt% with deionized water, and 5mL of pyrrole monomer (Aladdin, Shanghai, China) was added into 50 mL of the diluted solution with intensive mixing. The graphite fiber electrode (3 cm × 4 cm) was immersed in the above solution under continuous stirring, and then 10 mL of ammonium persulfate solution (1.92 g, Aladdin, Shanghai, China) as initiator was slowly added. Next, the obtained graphite fiber electrode (GF, (Hunan Yinfeng Co., Ltd., Changsha, China) and pulpy products were transferred to Teflon-lined autoclave (100 mL) after low temperature polymerization at 4 °C for 12 h. After hydrothermal reaction in 100 °C oven for 5 h, the GF was taken out and cleaned with deionized water, and then dried in 80 °C oven for 12 h. After that, the GF was placed in the tube furnace (OTF-1200X, Hefei, China) with argon as the protective gas, and the carbonization temperature was set to 800 °C, 900 °C and 1000 °C for 2 h, respectively. Finally, the resulting GF was cleaned with deionized water three times and dried at 80 °C to constant weight. The target electrodes were named GF@PCNs-800, GF@PCNs-900 and GF@PCNs-1000 according to the carbonization temperature. For contrastive analysis, the original GF was treated by the same process of GF@PCNs-900 without adding phytic acid solution, which is named GF@CG-900.

### 2.2. Structural Characterization

The surface morphology of the electrode material was characterized by scanning electron microscopy (SEM, SU8020, Japan) operated at 10 kV with energy dispersive spectroscopy (EDS) for elemental analysis. The graphitization degree and defects of the electrode material were studied by X-ray diffraction spectra (XRD, D/max 2500, Japan) and Raman spectra (Lab RAM HR Evolution, France) with a 532 nm laser excitation. The X-ray photoelectron spectroscopy (XPS, ESCALAB250XI, USA) was used to obtain the elemental composition, valence, and relative content of the electrode materials.

### 2.3. Electrochemical Measurements

The cyclic voltammetry test (CV) was performed on the electrochemical workstation (CHI760D, Shanghai, China), which was applied to analyze the electrode reaction process via a three-electrode system [5] in the 0.1 M VOSO_4_ + 3 M H_2_SO_4_ electrolyte, and the GF electrode (0.5 cm× 0.5 cm), platinum and silver chloride (Ag/AgCl) electrodes were used as the working electrode, the counter, and reference electrodes, respectively. The charge transfer impedance and ion diffusion resistance in electrolyte were analyzed by the impedance experiments (EIS) with a frequency range of 0.01–100 kHz at an amplitude of 5 mV under the same test condition as CV. The galvanostatic charging and discharging tests on a battery test system (Land CT2001A, Wuhan, China) were used to evaluate the overall performance of the VRFBs stack, and the design/structure of the flow battery is demonstrated in Figure S1, Supplementary Materials. The GF or GF@PCN-900 (2 × 2 cm^2^) served as both positive and negative electrodes separated by Nafion 115 (DuPont, Wilmington, DE, USA) as the membrane, and the copper foil and graphite plate were used as current collectors (6 × 6 cm^2^). The electrolytes at both sides were 0.75 M VOSO_4_ + 0.375 M V_2_(SO_4_)_3_ + 3 M H_2_SO_4_ (15 mL) (Hunan Yinfeng Co., Ltd., Changsha, China), and the flow rate was 30 mL min^−1^. The voltage window was set as 1.65-0.8V at the current density range of 100–400 mA cm^−2^, and the stability of the cell was verified by a long cycle test at a current density of 200 mA cm^−2^.

## 3. Results

**Figure 1:**
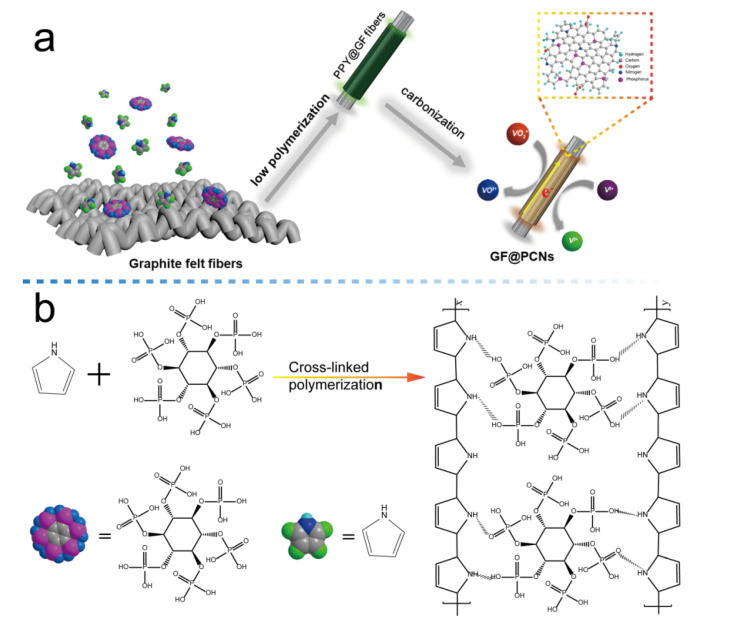
(**a**) The preparation process of GF@PCNs electrode, (**b**) Schematic diagram of cross-linking reaction on the surface of GF@PCNs.

As shown in Figure 1a, the GF@PCNs electrode was fabricated via low temperature polymerization between linear polymer monomer (pyrrole molecule) and phytic acid served as a cross-linking agent, and subsequent high-temperature carbonization on the GFs. Figure 1b demonstrated the schematic diagram of cross-linking reaction on the surface of GF@PCNs. The polypyrrole with linear polymer was obtained by the addition polymerization of pyrrole monomer, and the formed 3D nanonetwork structure was attributed to the hydrogen bond between the amino group in the polypyrrole chains and the phosphorus-oxygen functional group in the phytic acid molecule. In addition, the introduction of amino group and phosphorus-oxygen functional group, the oxygen, nitrogen and phosphorus elements were successfully doped into the composite electrodes, which is conducive to boost the electrochemical activity of reactive sites on the GF@PCNs for vanadium ions redox reaction.

**Figure 2:**
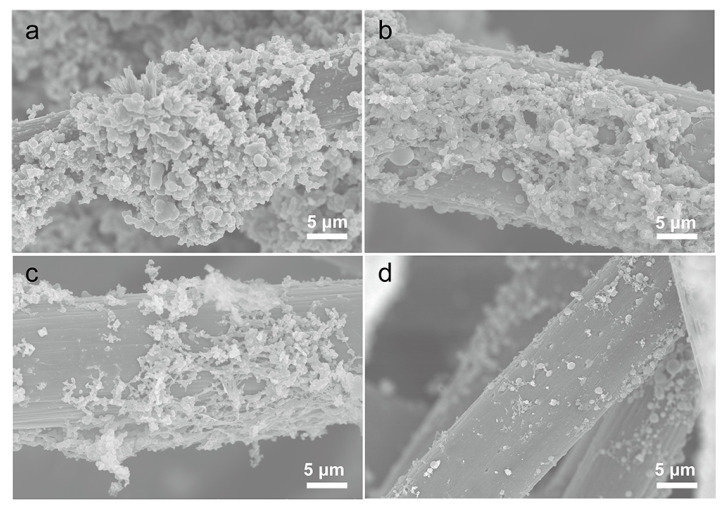
The SEM images of GF@PCNs electrode with various carbonized temperatures, (**a**) without carbonized process, (**b**) 800 °C, (**c**) 900 °C and (**d**) 1000 °C.

**Figure 3:**
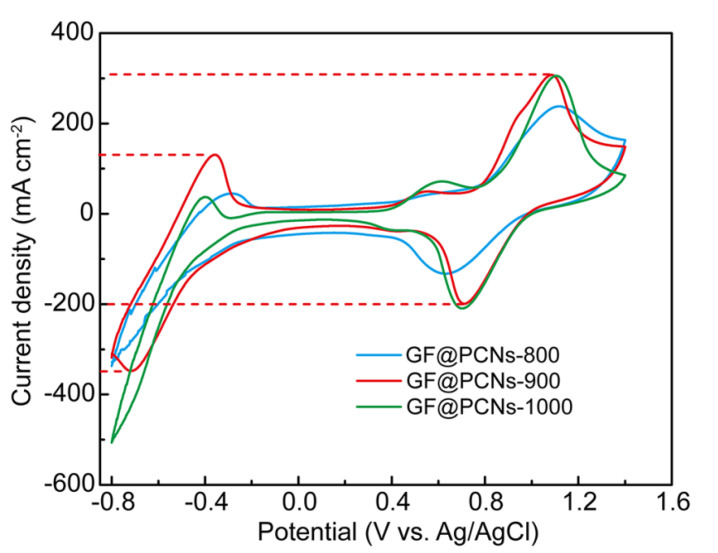
CV curves of GF@PCNs-800, GF@PCNs-900 and GF@PCNs-1000.

In order to analyze the effect of carbonization temperature on the surface structure of GFs, the scanning electron microscopy (SEM) was used to characterize the composite electrodes prepared at different carbonization temperatures. Figure 2a shows the surface structure of the composite electrode after low temperature polymerization and hydrothermal process without high temperature carbonization, and it can be seen that the moss-like carbon plates were distributed on the surface of the GF, indicating that the cross-linking polymerization was successfully realized. As show in Figure 2b, the surface of GF@PCNs-800 via carbonization at 800 °C was coated with a thick network structure with small pore size, by contrast, a 3D cross-linking network structure with developed porosity was formed on the electrode surface after carbonization at 900 °C (Figure 2c), while the network structure disappeared on the composite electrode surface after carbonization at a higher temperature of 1000 °C (Figure 2d). This difference in morphology suggests that low carbonization temperature is not enough to generate rich porous network structure on the GFs, and the generated network structure is apt to collapse under the excessive temperature. Therefore, the suitable carbonization temperature of 900 °C can effectively keep a well structure of 3D nanonetwork on the GF surface. To further verify the adaptability of carbonization temperature, the electrochemical performance of the composite electrode prepared at different carbonization temperatures was analyzed by cyclic voltammetry test (CV). The ratio of redox peak current (I_pc_/I_pa_) and the peak potential difference (ΔE = E_pa_ − E_pc_) can be applied to evaluate the reversibility and electrochemical polarization of vanadium ion redox reaction on different electrodes. As shown in Figure 3, the GF@PCNs-900 possessed the higher electrical conductivity and smaller electrochemical polarization for redox reaction compared with GF@PCNs-800 and GF@PCNs-1000, especially for the V^2+^/V^3+^ redox couple on the negative side. This result is attributed to the fact that lower carbonization temperature reduces the degree of graphitization, which affects the electrical conductivity of the composite electrode, but excessive temperature could instead lead to the partial reduction of the active functional groups, decreasing the electrocatalytic activity of the electrode materials.

**Figure 4:**
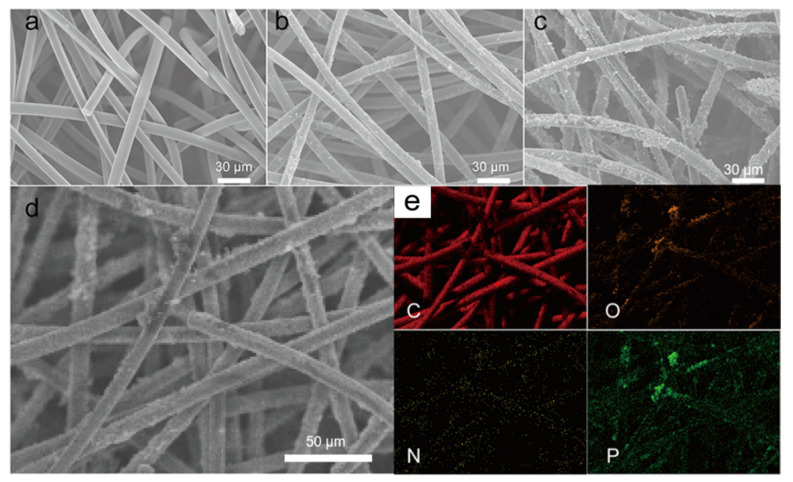
The SEM images of the (**a**) GF, (**b**) GF@CG-900, (**c**) GF@PCNs-900 and (**d**) the morphology and (**e**) element distribution of GF@PCNs-900.

On the basis of the above analysis, the effect of phytic acid molecule on the structure and property of GF@PCNs-900 was also studied. As shown in Figure 4a–c, compared with the smooth surface of the pristine GF, the GF@PCNs-900 is uniformly covered with nanonetwork structure, while the surface of GF@CG-900 is simply loaded with fewer carbon particles. The result exhibits the pyrrole monomer via polymerization and carbonization was transform into the zero-dimensional carbon particle on the surface of GF, and the phytic acid molecule served as cross-linking agent can effectively prompt the formation of 3D nanonetwork structure on the GF during high temperature carbonization. The constructed 3D conductive network is beneficial to improving the interface area between electrode and electrolyte, increasing the reaction zone for charge transfer and ion diffusion at the surface. In addition, it can be observed that carbon, oxygen, nitrogen, and phosphorus elements are evenly distributed on the surface of the GF@PCNs-900, as shown by energy-dispersive spectroscopy (EDS) (Figure 4d,e).

**Figure 5:**
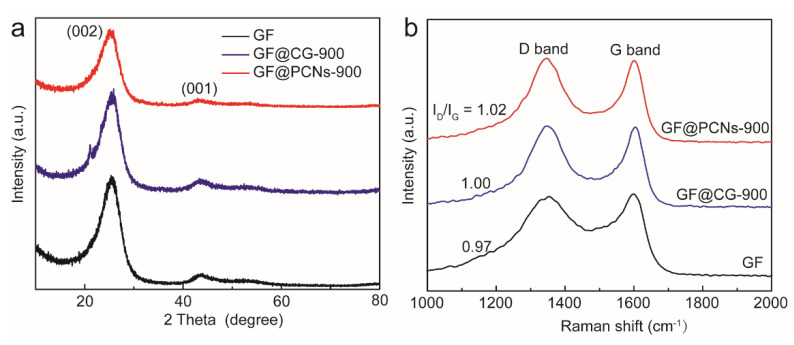
(**a**) XRD and (**b**) Raman spectrums of GF, GF@CG-900 and GF@PCNs-900.

The lattice defects of GF, GF@CG-900 and GF@PCNs-900 are analyzed by XRD. As shown in Figure 5a, the X-ray diffraction pattern shows two strong peaks at the positions of 26.5° and 44.0°, which belongs to the diffraction peaks of crystal plane (002) and (001) of graphite lattice. Compared with GF and GF@CG-900, GF@PCNs displays weaker peak intensity and larger half-peak width, indicating more defect sites on the composite electrodes causing by the multi-heteroatom doping. Similarly, the surface defects of different electrode materials were also detected by Raman spectroscopy. There are two Raman signals of 1584 cm^−1^ (G band) and 1340 cm^−1^ (D band) in the carbon materials, which ascribes the in-plane vibrations of sp2 carbon atoms and disordered structures, respectively, and the intensity ratio of the G and D bands (I_D_/I_G_) can be used to estimate the defect level of all samples. Figure 5b displays that the I_D_/I_G_ values of GF, GF@CG-900 and GF@PCNs-900 are 0.97, 1.00 and 1.02, respectively, implying that more surface defects are present in the GF@PCNs-900. The resulting defect sites can effectively increase the active sites and charge transfer efficiency for vanadium ion redox reaction, greatly boosting the electrocatalytic activity of GF@PCNs-900.

**Figure 6:**
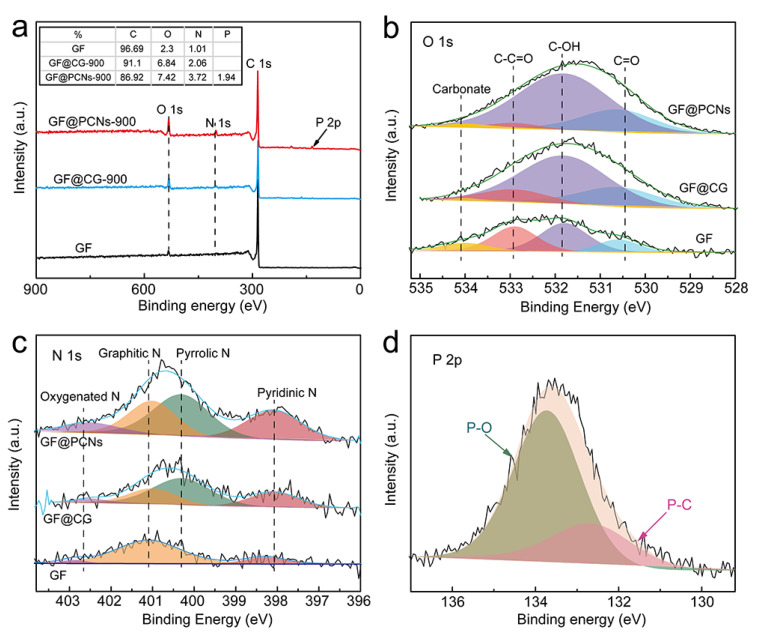
(**a**) XPS survey, (**b**) High-resolution XPS O 1s and (**c**) N 1s spectrum of the GF, GF@CG-900 and GF@PCNs900, (**d**) High-resolution XPS P 2p spectrum of GF@PCNs.

The element composition and relative content of the electrode material were analyzed by X-ray photoelectron spectroscopy (XPS). As shown in Figure 6a, the characteristic peaks of C 1s, O 1s and N 1s appeared in the GF and GF@CG-900 electrodes. In comparison, the GF@PCNs-900 possessed extra characteristic peaks of P 2p and P 2s, and the relative contents of oxygen and nitrogen were increased, indicating that the multi-element doping of phosphorus, nitrogen, and oxygen was successfully achieved at GF@PCNs-900. Figure 6b shows the O 1s spectra of various electrode materials. It can be observed that the characteristic peaks of GF, GF@CG-900, and GF@PCNs-900 electrodes appear at the positions of 530.6eV, 531.8eV, 532.9eV, and 534 eV, which are attributed to C=O, C-OH, C-C=O and carbonate, respectively [52]. The relative content of C-OH functional groups on the surface of GF@PCNs electrode is much higher than that of GF and GF@CG-900 electrode, and abundant C-OH functional groups can effectively elevate the redox reaction of vanadium ions. As shown in N 1s spectrum, the main forms of nitrogen in the electrode material are divided into oxygenated N (402.5 eV), graphitic N (401.0 eV), pyrrolic or pyridonic N (400.3 eV) and pyridinic N (398.2 eV), respectively [34] (Figure 6c). According to previous research, the pyrrole nitrogen and pyridine nitrogen has been considered as a more effective electrocatalytic activity center for vanadium ion redox reaction compared with other N species [53]. It can be seen that the increased nitrogen content of GF@PCNs-900 is mainly derived from the enhancement of pyrrolic or pyridonic N and pyridinic N, which suggests the nitrogen doping in this work is high-efficiency. In addition, Figure 6d demonstrates the high-resolution XPS P 2p spectrum of GF@PCNs-900, and the phosphorus element mainly existed in the form of P-O and P-C, located at 133.8eV and 132.7eV, respectively [54]. Owing to larger atomic radius, phosphorus doping can effectively break the π bond conjugated system among graphite carbon atoms and introduce more defect sites around neighboring carbon atoms, which is conductive to promoting the electrocatalytic activity of GF@PCNs-900 for vanadium ion redox reaction.

**Figure 7:**
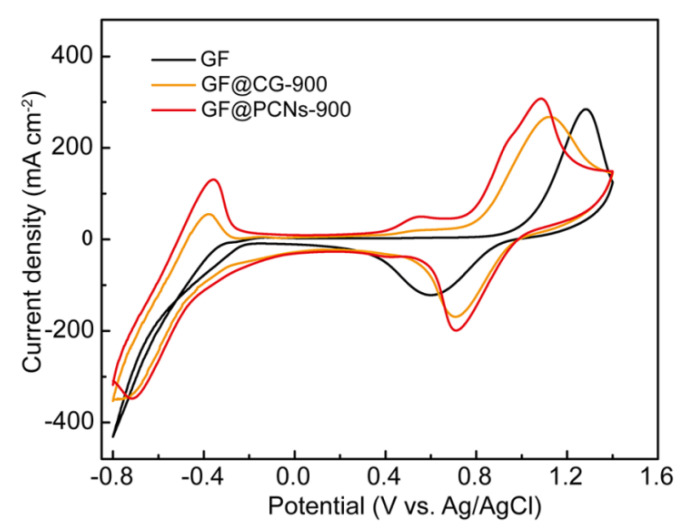
CV curves of GF, GF@CG-900 and GF@PCNs-900 at a scan rate of 10 mV s^−1^.

To analyze the electrode reaction process of various electrodes, CV curves were tested and shown in Figure 7. During the positive electrode reaction, compared with GF and GF@CG-900, the GF@PCNs-900 not only possessed a higher oxidation and reduction peak current and their ratio closer to 1, but also exhibited a smaller peak voltage difference, indicating that GF@PCNs-900 has an excellent electrocatalytic activity for the VO^2+^/VO_2_^+^ couple. Additionally, there is no reversible redox peak on GF electrode during the negative reaction, while GF@PCNs-900 showed a pair of obvious redox peaks and higher peak currents than GF@CG-900, which suggests the electrochemical activity of V^2+^/V^3+^ couple has been significantly improved. This outstanding performance is mainly due to that the 3D nanonetwork structure on GF@PCNs-900 can increase the contact area between the electrode and the electrolyte, facilitating the electron and ion conduction on the electrode surface. Meanwhile, multi-heteroatom doping can effectively enhance the electrochemical activity of the reaction site.

**Figure 8:**
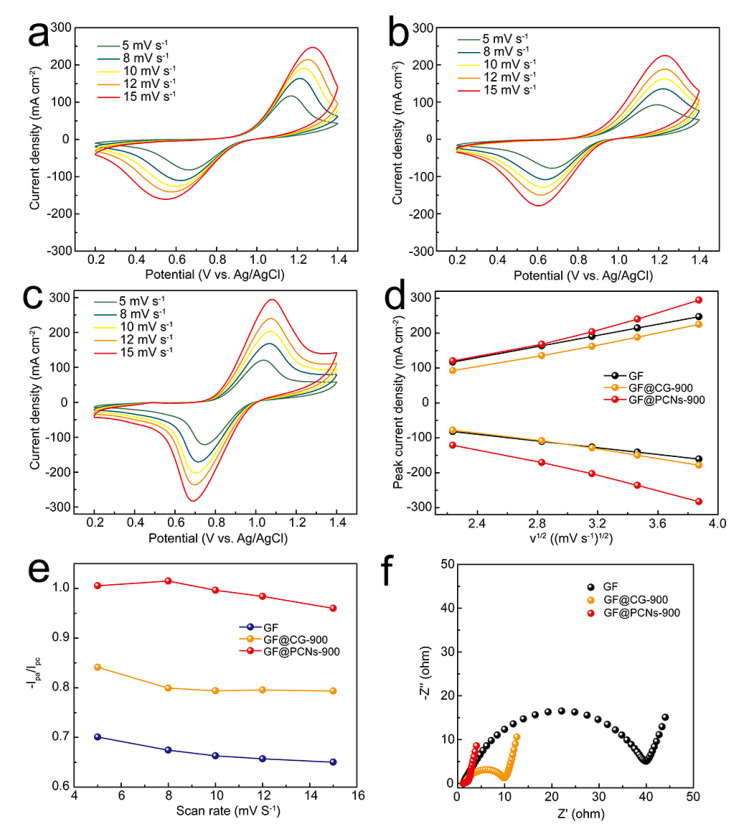
Multi-sweep tests of (**a**) GF, (**b**) GF@CG-900, (**c**) GF@PCNs-900 at various rate of 5, 8, 10, 12 and 15 mV/s, (**d**) the relationship curve between the square root of scan rate and peak current density, (**e**) the relationship between the peak current ratio and scan rate, (**f**) the Nyquist plots of the GF, GF@CG-900 and GF@PCNs-900.

The ion diffusion ability in the electrodes was analyzed by multi-sweep CV curves. Figure 8a–c displayed the CV curves of GF, GF@CG-900, and GF@PCNs-900 electrodes at scan rate of 5, 8, 10, 12, and 15 mV s^−1^, respectively. The relationship curve between the square root of the scan rate and peak current density is shown in Figure 8c, and their good linear relationship manifests the diffusion process known to control the electrochemical reaction of the VO^2+^/VO_2_^+^ couple. Based on Randles-Sevcik formula [55], it is known that the ionic diffusion coefficient is able to be reflected by the absolute value of slope of the relationship curve between the square root of scan rate and peak current density. No matter the oxidation or reduction process, the GF@PCNs-900 possessed a significantly higher slope value of the curve than that of GF and GF@CG-900 (Figure 8d), which highlights an outstanding vanadium ion diffusion during redox reaction. Moreover, at different scan rates, the I_pc_/I_pa_ of GF@PCNs-900 is closer to 1 compared with other electrodes, suggesting a high reversibility of the vanadium ion redox reaction (Figure 8e). Furthermore, the impedance experiments (EIS) were used to study the charge transfer impedance and ion diffusion resistance for the composite electrodes. As shown in Figure 8f, the GF@PCNs-900 exhibited a much lower charge transfer impedance of 2.06ohm than that of GF (40.07 ohm) and GF@CG-900 (10.20 ohm). The accelerated charge transfer largely originated from the fact that the 3D nanonetwork of the GF@PCNs-900 electrode facilitated the conduction of electrons and ions at the interface, and the multi-heteroatom doping improved the electrocatalytic activity of electrodes for the redox reaction.

**Figure 9:**
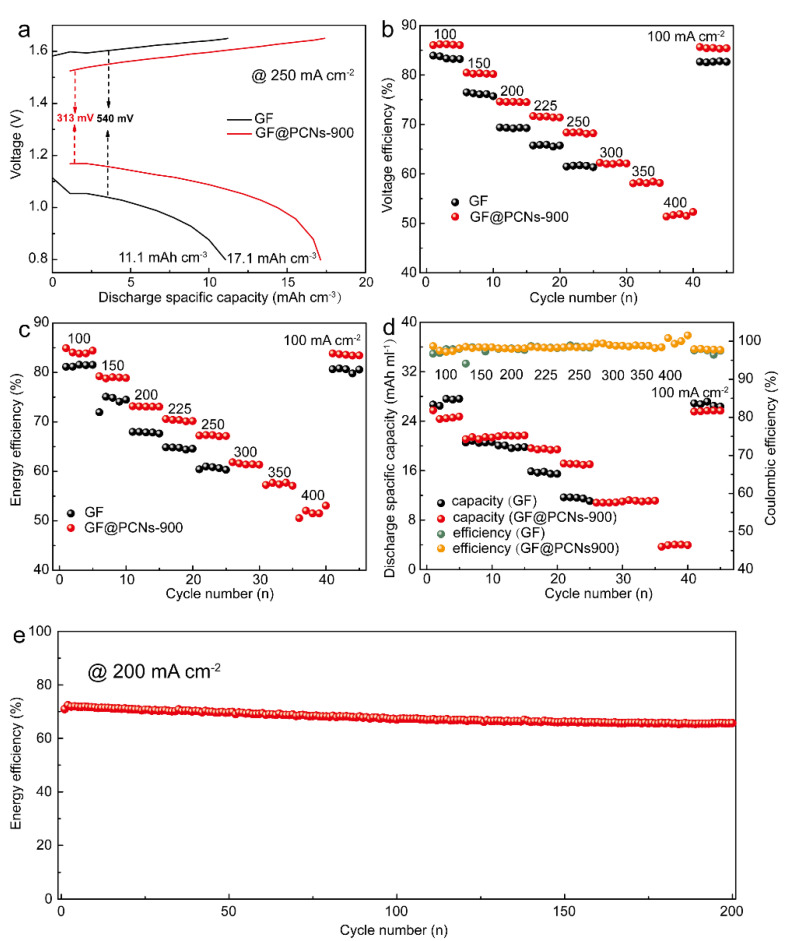
(**a**) Charge-discharge curve of the GF and GF@PCNs, (**b**) voltage efficiency, (**c**) energy efficiency, (**d**) discharge specific capacity and (**e**) cycling performances of GF@PCNs electrodes at 200 mA cm^−2^.

In order to analyze the electrochemical property of full batteries using various electrodes, GF and GF@PCNs-900 electrodes were assembled into VRFBs for the charge-discharge performance test, respectively (Figure 9). Figure 9a demonstrates the charge-discharge curve of the GF and GF@PCNs at the current density of 250 mA/cm^2^. Compared with pristine GF, the GF@PCNs possessed a higher discharging plateau and lower charging plateau, and the over potential of GF@PCNs-900 was decreased by 42.0%, and the discharge capacity was increased by 54.1%, indicating a small polarization effect during the charge-discharge process. In addition, the voltage efficiency (VE) can also reflect the polarization effect of VRFBs, which is calculated by the formula (VE = EE/CE). The EE and CE are energy efficiency and coulombic efficiency of VRFBs during charge/discharge process, respectively. As shown in Figure 9b,c, the GF@PCNs-900-based on VRFBs exhibited higher voltage efficiency (VE) and energy efficiency (EE) than GF-based on VRFBs at various current density, and the VE and EE of GF@PCNs-900-based on VRFBs has been enhanced by 12.1% and 11.6%, respectively, suggesting an excellent electrocatalytic activity of electrodes. In addition, the discharge specific capacity of GF and GF@PCNs-900 are displayed in Figure 9d, the GF-based on VRFBs exhibited the maximum current density of only 250 mA cm^−2^, while GF@PCNs-900 still maintained a discharge capacity of 5.0 Ah L^−1^ at a huge current density of 400 mA cm^−2^, surpassing most of the reported works (Table S1) [7,28,31,41,42,43,47,56,57,58]. Figure 9e shows the long cycle performance test of GF@PCNs-900-based on VRFBs at the current density of 200 mA cm^−2^. After 200 charge-discharge cycles, the energy efficiency of the GF@PCNs-900-based on VRFBs still maintains the initial value of 91.0%. The excellent rate performance and cyclic stability of GF@PCNs-900 are resulted from the enhancement of electron and ion transport ability at the 3D nanonetwork coated electrode interface, and the multi-heteroatom doping contributed to the improved electrochemical activity of reaction sites for vanadium ion redox reaction.

## 4. Conclusions

In this work, 3D nanonetwork coated GFs with multi-heteroatom doping were prepared for application in VRFBs via low temperature polymerization between linear polymer monomer and phytic acid, and subsequent carbonization (900 °C) on the GFs (GF@PCNs-900). Phytic acid played a role in stabilizing the 3D nanonetwork structure, and effectively realized the high-efficiency doping of nitrogen, oxygen, and phosphorus elements. Via the carbonization temperature comparison for CF@PCNs, it is known that the suitable carbonization temperature of 900 °C can effectively keep a well structure of 3D nanonetwork on the GF surface, and the GF@PCNs-900 possessed a higher electrical conductivity and smaller electrochemical polarization for the redox reaction. Their excellent electrochemical performance is mainly due to the fact that the 3D nanonetwork is beneficial to the sufficient diffusion of vanadium ions and rapid electron conduction along the GFs surface, and multi-heteroatom doping highly improves the electrocatalytic activity of composite electrodes toward the vanadium ions redox reaction. Finally, the VRFBs based on GF@PCNs delivered a higher energy efficiency of above 70% during 200 cycles at 200 mA cm^−2^ and an excellent rate performance for the discharge capacity of 5.0 Ah L^−1^ at a huge current density of 400 mA cm^−2^, surpassing most of the reported works. Therefore, the superior performance and materials designed in this work will advance the fundamental research and commercialization of VRFBs for large-scale energy storage.

## Data Availability

The data presented in this study are available on request from the corresponding author.

## Supplementary Materials

The following supporting information can be downloaded at: https://www.mdpi.com/article/10.3390/polym14235269/s1, Figure S1: The design/structure of the flow battery, Table S1: Comparison of the rate capability of the GF@PCNs electrode with previous work on electrodes materials for VRFBs. [7,28,31,41,42,43,47,56,57,58].
