# Supplementary material for: 3D Carbon Nanonetwork Coated Composite Electrode with Multi-Heteroatom Doping for High-Rate Vanadium Redox Flow Batteries
Source: Polymers (Basel). 2022 Dec 2;14(23):5269. doi: 10.3390/polym14235269 (PMC9738268; doi:10.3390/polym14235269)
Supplement: Supplementary file 1 [file polymers-14-05269-s001.zip › polymers-2044400-supplementary.pdf]

Supporting Information

3D Carbon Nanonetwork Coated Composite Electrode with Multi-heteroatom Doping for High-rate Vanadium Redox Flow Batteries

Wei Ling [1], Xiongwei Wu [2,*] and Funian Mo [1,*]

[1] School of Materials Science and Engineering, Harbin Institute of Technology (Shenzhen), Shenzhen 518055, PR China

[2] School of Chemistry and Materials Science, Hunan Agricultural University, Changsha 410128, P. R. China

* Correspondence: wxw@hunau.edu.cn; e-mail.com (X.W); mofunian@hit.edu.cn (F.M.)* E-mail: yanhuanglib@hit.edu.cn.

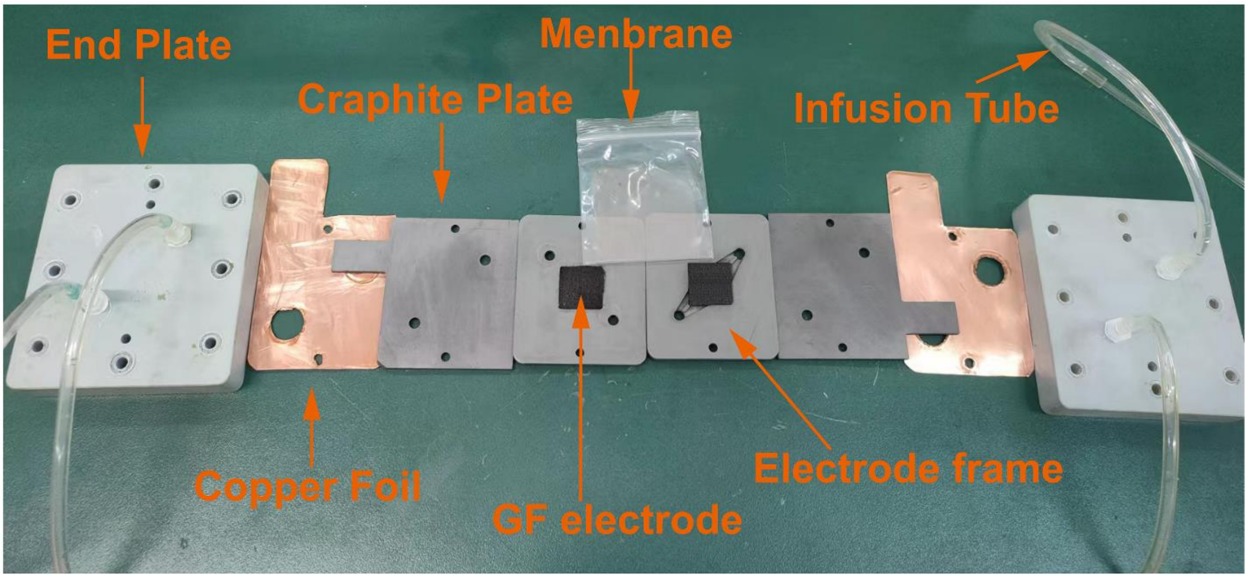

Figure S1. The design/structure of the flow battery.

Table S1. Comparison of the rate capability of the GF@PCNs electrode with previous work on electrodes materials for VRFBs

Reference	Electrode	Current density (mA cm^{-2})	Discharge capacity (Ah L^{-1})	Energy efficiency	Maximum Current density (mA cm^{-2})
**This work**	**GF@PCNs-900**	**250**<br>**400**	***17.1***<br>***5.0***	***68.0***<br>***54.0***	***400***
[1]Adv. Funct. Mater., 2019, 1903192	Exfoilated-GF	100<br>200	21.0<br>10.0	86.4<br>60.0	200
[2]J. Mater. Chem. A, 2019, 7, 5589-5600	$NiCoO_2$/GF	100<br>150	Not Given	73.7<br>72.5	150
[3]Nano Energy, 2018, 43, 55-62	PGF	150<br>200	28.0<br>24.0	73.0<br>68.0	300
[4]Energy Storage Mater., 2018, 13, 66-71.	GO-rGO/GF	50<br>100	Not Given	87.0<br>65.0	100
[5]J. Mater. Chem. A, 2018, 6, 6625-6632.	TiC-GF	80<br>100	26	67.0<br>62.0	100
[6]Chem. Eng. J., 2022, 450, 138377.	Bio-CF	100	Not Given	82	100
[7]Appl. Surf. Sci., 2021, 546, 148941.	B-CF	60<br>100	Not Given	85.0<br>80.0	300
[8]Nano Energy, 2016, 28, 19-28.	NCS/GF	150<br>200	27.0<br>23.0	71.0<br>67.0	300
[9] ChemNanoMat, 2022, 8, e202200027..	ASP-CF	100<br>200	Not Given	85.0<br>60.0	250
[10]Adv. Sci., 2016, 3, 1500276	CF-G-1	25<br>125	20.5	87.0<br>72.0	125

1. Mukhopadhyay, A.; Yang, Y.; Li, Y.; Chen, Y.; Li, H.; Natan, A.; Liu, Y.; Cao, D. and Zhu, H. Mass Transfer and Reaction Kinetic Enhanced Electrode for High‐Performance Aqueous Flow Batteries. *Adv. Funct. Mater.*, **2019**, 29, 1903192.
2. Xiang, Y. and Daoud, W. A. Binary Nicoo2-Modified Graphite Felt as an Advanced Positive Electrode for Vanadium Redox Flow Batteries. *J. Mater. Chem. A*, **2019**, *7*, 5589-5600.
3. Liu, Y.; Shen, Y.; Yu, L.; Liu, L.; Liang, F.; Qiu, X. and Xi, J. Holey-Engineered Electrodes for Advanced Vanadium Flow Batteries. *Nano Energy*, **2018**, *43*, 55-62.
4. Hu, G.; Jing, M.; Wang, D.-W.; Sun, Z.; Xu, C.; Ren, W.; Cheng, H.-M.; Yan, C.; Fan, X. and Li, F. A Gradient Bi-Functional Graphene-Based Modified Electrode for Vanadium Redox Flow Batteries. *Energy Storage Mater.*, **2018**, *13*, 66-71.
5. Ghimire, P. C.; Schweiss, R.; Scherer, G. G.; Wai, N.; Lim, T. M.; Bhattarai, A.; Nguyen, T. D. and Yan, Q. Titanium Carbide-Decorated Graphite Felt as High Performance Negative Electrode in Vanadium Redox Flow Batteries. *J. Mater. Chem. A*, **2018**, *6*, 6625-6632.
6. Hu, Z.; Miao, Z.; Xu, Z.; Zhu, X.; Zhong, F.; Ding, M.; Wang, J.; Xie, X.; Jia, C. and Liu, J. Carbon Felt Electrode Modified by Lotus Seed Shells for High-Performance Vanadium Redox Flow Battery. *Chem. Eng. J.*, **2022**, *450*, 138377.
7. Park, S. E.; Yang, S. Y. and Kim, K. J. Boron-Functionalized Carbon Felt Electrode for Enhancing the Electrochemical Performance of Vanadium Redox Flow Batteries. *Appl. Surf. Sci.*, **2021**, *546*, 148941.
8. Wu, L.; Shen, Y.; Yu, L.; Xi, J. and Qiu, X. Boosting Vanadium Flow Battery Performance by Nitrogen-Doped Carbon Nanospheres Electrocatalyst. *Nano Energy*, **2016**, *28*, 19-28.
9. Zhou, H.; Shen, Y.; Xi, J.; Qiu, X. and Chen, L. Zro2-Nanoparticle-Modified Graphite Felt: Bifunctional Effects on Vanadium Flow Batteries. *ACS Appl. Mater. Interfaces*, **2016**, *8*, 15369-15378.
10. Li, W.; Zhang, Z.; Tang, Y.; Bian, H.; Ng, T. W.; Zhang, W. and Lee, C. S. Graphene-Nanowall-Decorated Carbon Felt with Excellent Electrochemical Activity toward Vo2+/Vo2+ Couple for All Vanadium Redox Flow Battery. *Adv. Sci. (Weinh)*, **2016**, *3*, 1500276.
